# Phase-IIa randomized, double-blind, sham-controlled, parallel group trial on anodal transcranial direct current stimulation (tDCS) over the left and right tempo-parietal junction in autism spectrum disorder—StimAT: study protocol for a clinical trial

**DOI:** 10.1186/s13063-021-05172-1

**Published:** 2021-04-06

**Authors:** Christina Luckhardt, Magdalena Schütz, Andreas Mühlherr, Hannah Mössinger, Sara Boxhoorn, Astrid Dempfle, Ricardo Salvador, Giulio Ruffini, Helena C. Pereira, Miguel Castelo-Branco, Marianne Latinus, Frédérique Bonnet-Brilhault, Julia Siemann, Michael Siniatchkin, Christine Ecker, Christine M. Freitag

**Affiliations:** 1 Department of Child and Adolescent Psychiatry, Psychosomatics and Psychotherapy, University Hospital Frankfurt, Goethe University, Deutschordenstr.50, 60528 Frankfurt, Germany; 2 Institute of Medical Informatics and Statistics (IMIS), Kiel University, Brunswiker Str. 10, 24105 Kiel, Germany; 3 Neuroelectrics SLU, Av. Tibidabo 47 Bis, 08035 Barcelona, Spain; 4 Coimbra Institute for Biomedical Imaging and Translational Research (CIBIT), ICNAS, Faculty of Medicine, Academic Clinical Centre, University of Coimbra (UC), Paco das Escolas, 3001 451 Coimbra, Portugal; 5 UMR 1253, iBrain, Université de Tours, Inserm, Centre de Pédopsychiatrie, CHRU Bretonneau, 2 bd Tonnellé, 37044 Tours Cedex 9, France; 6 Centre Hospitalier Universitaire de Tours (CHUT), Centre Universitaire de Pédopsychiatrie, UMR930 INSERM / Equipe autism, CHRU Tours / Hôpital Bretonneau, 2 Bd Tonnellé, 37044 Tours Cedex 9, France; 7 Clinic of Child and Adolescent Psychiatry and Psychotherapy, Protestant Hospital Bethel, EvKB, Remterweg 13a, 33617 Bielefeld, Germany

**Keywords:** Autism spectrum disorder, Transcranial direct current stimulation, tDCS, Randomized controlled trial, Tempo-parietal junction

## Abstract

**Background:**

Autism spectrum disorder (ASD) is characterized by impaired social communication and interaction, and stereotyped, repetitive behaviour and sensory interests. To date, there is no effective medication that can improve social communication and interaction in ASD, and effect sizes of behaviour-based psychotherapy remain in the low to medium range. Consequently, there is a clear need for new treatment options. ASD is associated with altered activation and connectivity patterns in brain areas which process social information. Transcranial direct current stimulation (tDCS) is a technique that applies a weak electrical current to the brain in order to modulate neural excitability and alter connectivity. Combined with specific cognitive tasks, it allows to facilitate and consolidate the respective training effects. Therefore, application of tDCS in brain areas relevant to social cognition in combination with a specific cognitive training is a promising treatment approach for ASD.

**Methods:**

A phase-IIa pilot randomized, double-blind, sham-controlled, parallel-group clinical study is presented, which aims at investigating if 10 days of 20-min multi-channel tDCS stimulation of the bilateral tempo-parietal junction (TPJ) at 2.0 mA in combination with a computer-based cognitive training on perspective taking, intention and emotion understanding, can improve social cognitive abilities in children and adolescents with ASD. The main objectives are to describe the change in parent-rated social responsiveness from baseline (within 1 week before first stimulation) to post-intervention (within 7 days after last stimulation) and to monitor safety and tolerability of the intervention. Secondary objectives include the evaluation of change in parent-rated social responsiveness at follow-up (4 weeks after end of intervention), change in other ASD core symptoms and psychopathology, social cognitive abilities and neural functioning post-intervention and at follow-up in order to explore underlying neural and cognitive mechanisms.

**Discussion:**

If shown, positive results regarding change in parent-rated social cognition and favourable safety and tolerability of the intervention will confirm tDCS as a promising treatment for ASD core-symptoms. This may be a first step in establishing a new and cost-efficient intervention for individuals with ASD.

**Trial registration:**

The trial is registered with the German Clinical Trials Register (DRKS), DRKS00014732. Registered on 15 August 2018.

**Protocol version:**

This study protocol refers to protocol version 1.2 from 24 May 2019.

**Supplementary Information:**

The online version contains supplementary material available at 10.1186/s13063-021-05172-1.

## Background

Autism spectrum disorder (ASD) is characterized by impairments in social communication and interaction, as well as stereotyped and repetitive behaviours and interests [1]. With a prevalence of 1% in children and adolescents of the European population [2], and a global increase in ASD prevalence over the past years [3], there is a strong need for effective interventions. The overall prognosis is currently poor, as only around 20% of the individuals with ASD are able to lead independent lives as adults [4]. Societal costs are high [5, 6], and quality of life is reduced in individuals with ASD [7].

To date, no effective pharmacotherapy for the core symptoms of impaired social interaction and communication has been developed. Early, behaviourally based intervention in infancy and toddlerhood has a medium effect on social reciprocity in ASD [8], and autism-specific social skills training can lead to small to medium improvements in social responsiveness in older children and adolescents with high-functioning ASD [9, 10]. Still, high variability in individual outcomes has been observed in most psychotherapeutic intervention studies, and overall effect sizes often remain in the small to medium range. Therefore, there is a need for new treatment options targeting the underlying neurobiological mechanisms of the disorder.

Brain stimulation techniques represent a new and promising alternative to medication and psychotherapy for the treatment of mental disorders [11]. Especially transcranial direct current stimulation (tDCS), which uses low-intensity electrical stimulation (0.5–2.0 mA) applied via anode and cathode electrodes placed on the surface of the scalp [12], is an innovative and cost-effective treatment approach. tDCS alters spontaneous neural activity. The positively charged current from the anode usually increases cortical excitability, while the negatively charged cathode usually decreases it [13]. This modulation is brought upon by a modification of the resting membrane potential in regions of current flow [14].

tDCS has been successfully employed to modulate resting state activity [15] as well as functional connectivity of brain networks [16]. The effects of tDCS on various perceptual, motor and cognitive processes have also been studied extensively. While single sessions of tDCS often lead to small and inconsistent effects, designs comprising several tDCS sessions lead to stronger and more consistent improvements [17]. Furthermore, meta-analytic evidence indicates that on-line stimulation (i.e. tDCS stimulation during performance of a cognitive task, which involves the stimulated area) leads to stronger improvements in cognitive performance, especially in neuropsychiatric populations such as children and adolescents with attention deficit hyperactivity disorder (ADHD [18]).

A recent systematic review indicated that tDCS is a highly promising treatment approach for children and adolescents with ASD, as it has the potential to ameliorate ASD-typical patterns of altered neural functioning including aberrant brain connectivity patterns [19]. In fact, several studies have already investigated the effects of tDCS over the dorsolateral prefrontal cortex (DLPFC) on autistic symptoms, electroencephalogram (EEG) resting state connectivity, executive function, working memory and syntax acquisition [20–26]. These results indicate that tDCS is a promising treatment approach for ASD and confirm favourable safety evaluations similar to those reported for typically developing children [27] and adults [28]. However, only small samples have been investigated with highly variable study designs and safety has not always been systematically evaluated. Furthermore, DLPFC functions are only indirectly linked to the core symptoms of social cognition and social communication impairments in ASD. Targeting brain areas which are directly involved in these processes therefore could be an even more promising approach.

Brain networks affected in ASD consist of structures such as the superior temporal sulcus (STS) and the temporo-parietal junction (TPJ). Especially the TPJ consistently shows patterns of decreased activation and connectivity in individuals with ASD [29, 30]. The TPJ includes several sub-regions such as the angular gyrus, lateral occipital cortex and supramarginal gyrus [31]. Together, these regions form a key hub within the “social brain” [32] that relates to many social cognitive functions which are affected in individuals with ASD, such as theory of mind [33], attention, visuo-motor processing, speech and language, self-other differentiation and social cognition [34–36]. tDCS over the TPJ has been shown to improve social cooperation, perspective taking and emotion attribution in healthy individuals [37–39].

To date, only one small-scale pilot study investigated the effect of a single session of anodal stimulation of the right TPJ at 2 mA with a concurrent skills training. Results, which are based on the behavioural data of six adults with high-functioning ASD, indicate higher verbal fluency following verum compared to sham stimulation, and a trend for improvement in a pre-post comparison of social skills in the verum group [40]. However, the small sample size, the application of tDCS for only a single session and the lack of validated clinical outcome measures highlight the preliminary nature of this study. More highly powered randomized controlled trials with carefully selected outcome measures are needed to accurately estimate the effect size of changes that can be elicited by tDCS to the TPJ.

In the presented protocol, we aim to investigate if 10 × 20 min multi-channel 2 mA anodal stimulation of the bilateral TPJ in children and adolescents with ASD, in addition to a computer-based cognitive training, will improve disorder-specific neurocognitive and behavioural impairments. The study is a phase-IIa pilot randomized, double-blind, sham-controlled, parallel-group clinical study, with the main objective to estimate effect sizes of change in parent-rated social responsiveness, and to monitor safety and tolerability of the intervention.

## Methods/design

### Aims and objectives

The study “StimAT” aims to investigate whether repeated (10 × 20 min) multi-channel 2 mA anodal stimulation of the bilateral tempo-parietal junction, in addition to a computer based cognitive training, will improve relevant behavioural and cognitive symptoms in children and adolescents with ASD aged 10 to < 18 years old compared to the combination of the computer-based training with sham stimulation.

#### Primary objectives

- To investigate the effect size of change in parent-rated social responsiveness total score (SRS-16-item short form) between baseline (T2, within 1 week before first stimulation) and post-intervention (T3, within 7 days after last stimulation) after 10 sessions of multi-channel anodal tDCS over bilateral TPJ with a concurrent cognitive training in children and adolescents with ASD compared to sham stimulation during the same cognitive training.
- To study safety and tolerability of multi-channel anodal tDCS targeting the TPJ in children and adolescents with ASD.

#### Secondary objectives

- To investigate effect size of change in parent-rated social responsiveness (SRS-16-item short form) between baseline (T2) and follow-up (T4, within 3–4 weeks after T3, within 4–5 weeks after last stimulation)
- To investigate effect sizes of change in ASD core symptoms and associated psychopathology (Repetitive Behaviour Scale-revised, RBS-R; Children’s Communication Checklist-2, CCC-2; all subscales of the Child Behaviour Checklist, CBCL; Aberrant Behavior Checklist, ABC) and all areas of health-related quality of life according to KIDSCREEN-27 (parent and child)
- To investigate effect sizes of change in imitation abilities of non-meaningful gestures [41] between baseline (T2) and end of treatment (T3) and baseline to follow-up (T4)
- To investigate effect sizes of change in social cognition (Cambridge Neuropsychological Test Automated Battery (CANTAB), Emotion Recognition Task error rates and reaction times) and attention (Reaction Time Test, Spatial Span Test, One Touch Stockings of Cambridge, error rates and reaction times) between baseline (T2) and end of treatment (T3) and baseline (T2) to follow-up (T4)
- To investigate effect sizes of change in reaction time and error rates of behavioural tasks assessing intentionality, visual perspective taking, emotion recognition and attention between baseline (T2) and end of treatment (T3) and baseline (T2) to follow-up (T4)
- To investigate effect sizes of change in neurophysiological measures (amplitude and latency, latency variability of evoked potentials, neuronal sources of task relevant components and oscillatory activity) assessed by EEG (tasks: intentionality, visual perspective taking, emotion recognition, Posner task) between baseline (T2) and end of treatment (T3) and baseline (T2) to follow-up (T4)
- To study structural and functional magnetic resonance imaging (MRI) measures at T2 as predictors of change in resting-state fMRI/EEG measures at T3
- To assess expectations and concerns of parents and ASD individuals towards tDCS

### Design

The study is a multi-centre, two-arm randomized, double-blind, parallel group, sham-controlled pilot phase IIa-trial with one time-point for screening/inclusion (T1) and three measurement points (T2, baseline; T3, post-intervention; T4, follow-up). Arm one is repeated anodal tDCS over bilateral TPJ (10 days of stimulation, 20 min/day, with 2 mA total injected current) during a concurrent computer-based cognitive training focussing on perspective taking, intention and emotion understanding. Arm two is the blinded sham stimulation of 10 × 20 min applied during the same computer-based training as the comparator.

**Fig. 1:**
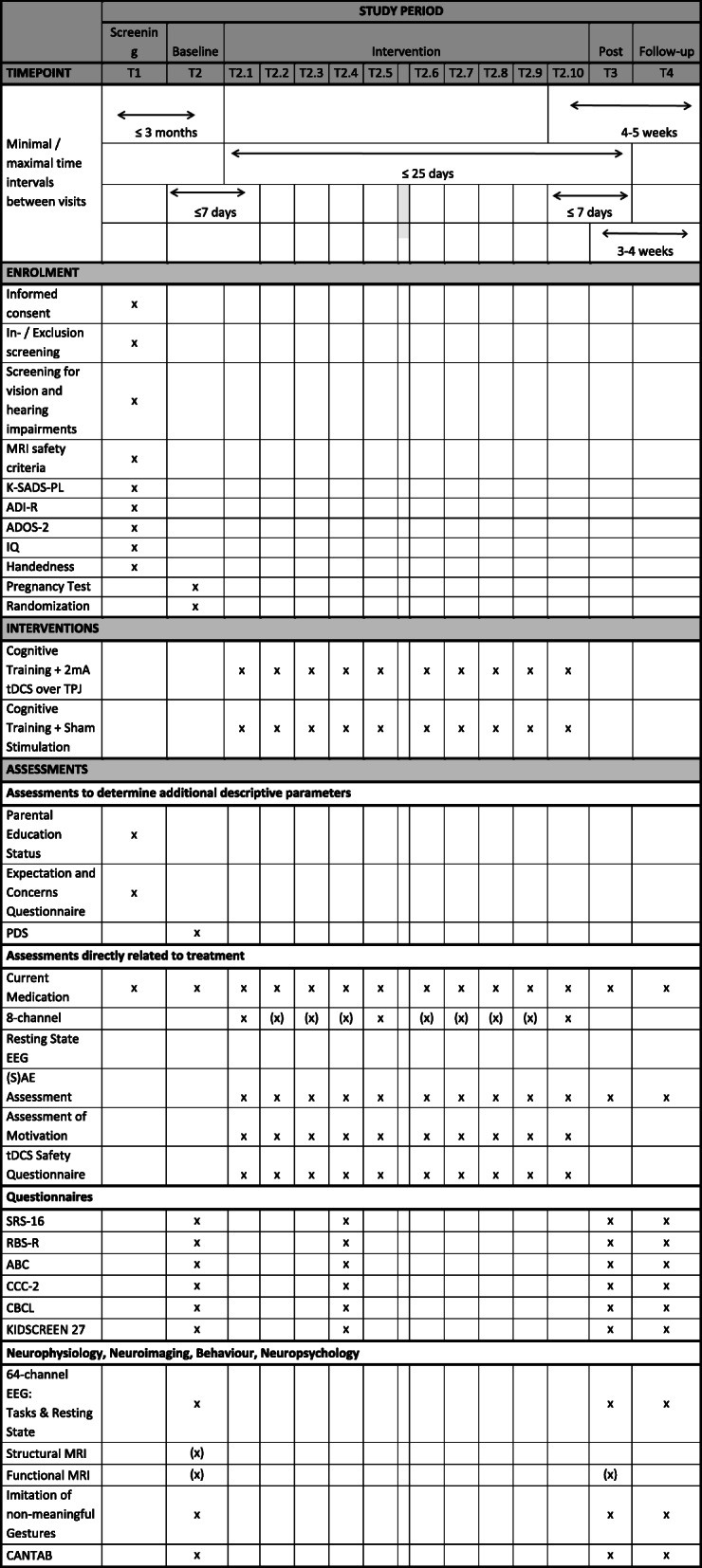
schedule of trial participation and overview of applied diagnostic and outcome measures

Timeline of study participation and collection of all outcome measures is summarized in Fig. 1. At visit T1 (screening), participants and parents are informed by a qualified study investigators about all aspects of the trial and are asked to give written informed consent/assent. Afterwards, the necessary information on inclusion and exclusion criteria is collected and assessed: developmental and medical history (including information on current medication and somatic or neurological disorders), screening for vision and hearing impairments, Autism Diagnostic Interview-Revised (ADI-R [42]), Autism Diagnostic Observation Schedule-2 (ADOS-2 [43]), Schedule for Affective Disorders and Schizophrenia for School-Age Children-Present and Lifetime Version (K-SADS-PL [44]), IQ-Test [45, 46], tDCS/MRI safety criteria and tDCS participation criteria (see supplement for further information). Information on the child’s and parents’ expectations and concerns towards tDCS are obtained by a questionnaire. At visit T2 (baseline, within 3 months after screening), a urine pregnancy test is performed with female participants, the pubertal development status (PDS [47]) is assessed, questionnaire and MRI data is collected and neurocognitive tests with concurrent EEG and eye-tracking are performed (see the “Outcome measures” section below).

Visits T2-1 to T2-10 represent the 10 days of intervention, over a 2-week period (usually in two blocks of 5 days with a 2-day break over the weekend; T2-1 within 7 days after T2, up to three missed interventions of T2-1 to T2-10 may be rescheduled in a 3rd week). Each session consists of stimulation (sham/active) for 20 min while the computer-based cognitive training is performed. At each intervention session, the possible occurrence of adverse events (AEs), serious adverse events (SAEs) and the current medication is documented; a tDCS safety questionnaire is collected; and motivation is assessed. Pre- and post-stimulation resting-state EEG (8-channel) is collected at T2-1, T2-5 and T2-10 (required; optionally resting state EEG can be collected pre and post each stimulation session). At T2-5 questionnaires are additionally collected (see Fig. 1).

The T3-post intervention assessment (within 7 days after T2-10, within 25 days after T2) includes (S)AE and current medication documentation, collection of questionnaires and MRI data as well as neurocognitive tests, EEG and eye-tracking measures (see the “Outcome measures” section below). The same data (except MRI) is again collected at T4 (follow-up).

Study participation ends with the last follow-up visit (T4). However, participants as well as their caregivers can withdraw from the study at any time and without providing reasons. Their concurrent or subsequent treatment will not be affected or compromised because of the decision to withdraw. Moreover, the investigator can decide to end participation in the clinical trial if there is reason to assume that the intervention is harmful for the participant. Furthermore participants, who are non-compliant to a degree that either their safety or the integrity of data is at risk, may be excluded from further treatment at the investigator’s discretion. Reasons for exclusion from the study will be documented in the eCRF.

### Outcome measures

The primary outcome measure is the effect size of change in parent-rated social responsiveness (SRS-16-item short form [48]) from baseline (T2) to post-intervention (T3). The SRS-16 is a short version of the Social Responsiveness Scale (SRS) [49] which combines the raw score of 16 items from the original SRS into a short version based on item response theory to measure autistic traits, particularly reciprocal social behaviour. The SRS-16 shows high reliability (*α* = .96) and strong correlations with the full length SRS (*r* = .98) as well as other measures of ASD symptom severity [48].

Ratings and observations of safety and tolerability of tDCS stimulation will be assessed at all intervention visits based on an established safety questionnaire [50, 51]. The questionnaire comprises 8 items regarding side effects such as itching, pain, burning, warmth, fatigue and other adverse effects. The participant is asked to rate the incidence/intensity on a 4-point Likert-scale (“none” = 0 to “strong” = 3), to indicate when and where (if localized) the side effect occurred, as well as how disturbing it felt (on a 5-point Likert scale from “not at all” to “extremely”). Furthermore, a descriptive comparison of (S) AEs during the entire study will be made between groups.

Secondary outcome measures obtained at baseline (T2), post-intervention (T3) and follow-up (T4) include the following:

- Repetitive Behaviour Scale-revised (RBS-R) total score and subscales. The parent-rated Repetitive Behaviour Scale-Revised (RBS-R, [52]) measures autism-specific repetitive behaviours. Total score and five subscales (stereotyped, self-injurious, compulsive, ritualistic and restricted behaviour) are derived. One-month test re-test reliability was *α* = 0.97 (total score, [53]). Internal consistency for the subscales is satisfying ranging from *α* = .78 to *α* = .91. High correlations with the CBCL and the ADI-R were found [54]
- Children’s Communication Checklist-2, CCC-2 [55]. The 70-item questionnaire completed by a parent screens for communication problems. Two composites are derived: The General Communication Composite (GCC) is used to identify children likely to have clinically significant communication problems; the Social Interaction Deviance Composite can assist in identifying children with a communicative profile characteristic of autism [55]. The GCC as well as factor scores derived from factor analysis are used as outcome.
- Child Behaviour Checklist (CBCL). The parent rating form the Child Behaviour Checklist (CBCL) [56] is one of the most widely used valid and reliable measures in clinical research, dimensionally measuring social-emotional or behavioural problems. It contains 99 items from which a total score, the internalizing/externalizing scores and 6 subscales are derived.
- Aberrant Behavior Checklist (ABC [57]). The factors of the Aberrant Behavior Checklist are as follows: (I) Irritability, (II) Lethargy, (III) Stereotypic Behavior, (IV) Hyperactivity, Noncompliance and (V) Inappropriate Speech. It is an empirically developed scale to measure psychiatric symptoms and behavioural disturbance exhibited by individuals with intellectual and developmental disability.
- All areas of health-related quality of life according to KIDSCREEN-27 (parent and child). The Kidscreen-27 is an instrument that assesses children’s and adolescents’ (ages 8–18 years) subjective health and well-being. It contains 27 items that cover five separate domains: physical well-being, psychological well-being, autonomy and parents, peers and social support and school environment. Two-week test–retest reliability is at 0.61 to 0.74 [58].
- Imitation abilities of non-meaningful gestures [41] are tested behaviourally by an examiner demonstrating non-meaningful hand, finger and hand-finger gestures which have to be reproduced by the participant. Either hand position relative to the head, finger configuration or both are assessed. Correct imitation after the first demonstration is scored 2 points, after the second demonstration 1 point. The test includes 14 items for hand position, 14 for finger configuration and 14 for combined hand and finger gestures [41].
- Cognitive tests by the CANTAB (“CANTAB® Cognitive assessment software”): (a) The One Touch Stockings of Cambridge, which examines executive functions and requires both spatial planning and working memory; (b) Spatial Span Test, which assesses visual working memory capacity; (c) the Reaction Time Test, assessing movement time, reaction time, response accuracy and impulsivity; and (d) the Emotion Recognition Task, which measures the ability to identify basic emotions in facial expressions. Error-rates, reaction times and span length will be assessed.
- Resting state EEG (4 min eyes open, 4 min eyes closed), behavioural performance (error rates and reaction times) and neurophysiological measures (amplitude, latency and latency variability of evoked potentials, neuronal sources of task relevant components and oscillatory activity) will be obtained by a 64-channel EEG in four tasks. Concurrent eye tracking is performed to record gaze shifts and pupil dilation. The skills needed for the tasks are associated with TPJ functioning and are known to be impaired in ASD: (a) Intentionality will be assessed in a task adapted from Vistoli et al. [59] in which comic scenes requiring the attribution of intent to a character or understanding of logical sequence of events based on physical causality are shown. (b) Visual perspective taking (adapted from [60, 61] will be assessed using the “dot perspective task”. (c) Emotion recognition will be assessed in a paradigm presented with a virtual avatar whose face morphs from a neutral expression to either “happy” or “sad” [62]. (d) A Posner task will be used to examine abilities of attention reorienting (adapted from [63, 64]).
- 8-channel resting-state EEG is also recorded before and after the first, fifth and last stimulation (T2-1, T2-5 and T2-10).
- At post-intervention (T2) and follow-up (T3), resting-state MRI will be obtained and a voice localizing task will be presented [65, 66] assessing voice perception.
- Furthermore, structural MRI and diffusion tensor imaging (DTI) data collected at baseline (T2) are evaluated to develop biomarkers to predict response to tDCS.

### Participants, inclusion and exclusion criteria

It is planned to include 100 participants with ASD between the ages of 10 and < 18 years old at randomization. Both boys and girls can participate in the study. However, in ASD populations, there is a sex distribution in favour of males, as boys are approximately 3 times more likely to be affected than girls [67]. Consequently, the gender distribution in the recruited sample is likely to reflect this population-based imbalance.

Inclusion criteria are an expert clinical ASD diagnosis according to DSM-5 supported by ADI-R and ADOS-2, including all Autism Spectrum Disorders (299.00), and the participant and their parents have to be able and willing to give written informed assent/consent.

Exclusion criteria are IQ < 70, birth weight < 2000 g, cerebral palsy, tuberous sclerosis, neurofibromatosis, history of brain surgery, cochlear implant, skull deformity, history of craniocerebral injury with loss of consciousness, increased intracranial pressure, history of epilepsy or seizures or migraine in patients or first-degree relatives, and current vision (less than 80% of normal or corrected to normal vision in both eyes) or hearing impairments. Any comorbid mental disorders other than the following (classified according to DSM-5): all communication disorders, attention deficit hyperactivity disorder (ADHD), specific learning disorder with IQ > =70, developmental coordination disorder, major depressive disorder (mild depressive episode, dysthymia), all anxiety disorders, all elimination disorders, gender dysphoria, oppositional defiant disorder, ASD-related sleeping problems and ASD-related eating problems. Furthermore dermatological disease of the scalp or chronic skin damage; any electrically, mechanically or magnetically activated implant (for example, brain implant, pacemakers, cardiac implants, vascular clips); heart disease; current pregnancy or hormonal contraception; and history of smoking within last 5 years are also excluded.

The following psychopharmacological and therapeutic treatment is permitted during study participation: any antidepressive medication (atomoxetine, guanfacine) and any antipsychotic medication with a stable dosage for at least 4 weeks prior to T2 until T4 and methylphenidate, any amphetamine preparation and melatonin with a stable dosage for at least 1 week prior to T2 until T4. Concurrent neurofeedback, stimulation therapy, or participation in other clinical trials is not allowed. Other ongoing interventions such as behavioural therapy or occupational therapy can be continued.

### Recruitment measures

Four sites with extensive experience and expertise in the investigation and treatment of children and adolescents with ASD participate in the trial: (1) the Department of Child and Adolescent Psychiatry, Psychosomatics and Psychotherapy and Autism Research and Intervention Center of Excellence at the University Hospital Frankfurt, Goethe University; (2) the Department of Child and Adolescent Psychiatry, Psychosomatics and Psychotherapy at Bethel Protestant Hospital in Bielefeld, Germany (3) the ICNAS, Centro Clínico Académico, Universidade de Coimbra, Portugal; and (4) the Centre Hospitalier Regional Universitaire de Tours, Tours, France. All sites are associated with large in- and outpatient departments, so families and adolescents with ASD can be approached and specifically invited to participate in the study. Additional patients will be approached via flyers and by announcements published in the local newspapers and on the internet. Local paediatricians, child and adolescent psychiatrists and psychotherapists as well as ASD patient and parent organizations will be informed about the study.

### Assignment to study arm / randomization

After definite inclusion into the study (signed informed consent, fulfilment of all inclusion criteria), each patient will be allocated randomly to one of the two treatment conditions (anodal tDCS stimulation or sham stimulation) via the electronic case report form (eCRF) using a stratified block randomization. Randomization will be stratified by site and gender. Block randomization for each stratum is prepared with the electronic randomization tool BiAS for Windows. Randomization is performed by Institute of Medical Informatics and Statistics (IMIS)/Center for Clinical Trials (ZKS) Kiel.

### Blinding

Subjects, parents and study personnel will be blinded to treatment allocation for the whole duration of the trial. The device Starstim32 (Neuroelectrics SLU, Barcelona, Spain) allows to perform double-blind stimulation. Prior to study start, an administrator at Neuroelectrics who can create and manage stimulation protocols creates templates for real and sham stimulation. Each centre receives a series of files from the statistician which do not reveal any information of the condition (tDCS or sham). At randomization, one specific file is assigned to each participant via the eCRF. During the stimulation session, the study personnel will have no access to the password-protected content of this file (protocol data, i.e. currents, sham-settings) and thus remain effectively blinded.

Unblinding will be done in case of the following emergencies: unblinding is necessary for reasons of subjects’ safety and for decisions on further medical treatment and a serious adverse device effect (SADE) has to be unblinded for notification to authorities and ethics committees. Unblinding will be performed through emergency envelopes on site, which contain information on sham or real stimulation. The participant and his/her parents must be informed about unblinding. Unblinding leads to the exclusion of the respective participant from further intervention sessions.

### Intervention

The intervention consists of 10 sessions of either tDCS- or sham stimulation during a socio-cognitive training. The real stimulation is anodal stimulation of the TPJ at 2 mA for a duration of 20 min (plus 30-s ramp-up and 30-s ramp-down) per session. The control intervention is a sham stimulation (at the beginning 30-s ramp-up followed by 30-s fade out after 5 s and 30 s fade in and then 30 s ramp-down at the end, but no effective stimulation for the remaining duration of the intervention), which also has a total duration of 21 min including ramp-up and ramp-down. This type of sham stimulation is routinely used as a comparison to actual tDCS stimulation. The short transient application of current at the beginning and at the end induces the sensory impression of being stimulated and serves to improve the blinding of participants, while no meaningful changes in neural excitability is induced. The medical device used for the intervention (Starstim 32®, Neuroelectrics) is a transcranial current stimulation and EEG monitoring device. An optimized multi-channel montage is used to target the TPJ bilaterally based on an area identified using DTI-based tractography [68]. The target area covers regions of the TPJ which were identified as functionally relevant for social cognitive abilities [31]. The optimization method used is the Stimweaver algorithm [69], which determines the electrode positions and currents that better approximate a target distribution of currents in a computational numerical head model (Colin head model, [70]). The optimized montage consists of two anode-electrodes, one over the left and right TPJ each, with several cathode-electrodes placed around each anode. The maximal injected current into the brain at any given time will be below 2.00 mA and maximum current at any electrode is 1.00 mA. The electrodes used in this study are 1 cm radius Ag/AgCl cylindrical electrodes with conductive gel underneath (NG PiStim electrodes).

Both, the tDCS and sham groups, will perform a cognitive training battery targeting theory of mind and perspective taking abilities during the intervention sessions. The training battery consists of short film clips, followed by questions probing the participant’s understanding of the character’s intentions, thoughts or feelings. Depending on whether the participant chooses a correct or incorrect answer to the question, the participant will either have to explain how they knew the correct answer, or receive an opportunity to repeat the scene and question if an incorrect answer was given. Before the film clip is repeated, they will receive a prompt about what to attend to in order to answer the question correctly. If they do not succeed on the second attempt, detailed feedback will be presented to ensure the participant understood which answer was correct and why. This detailed feedback involves highlighting the salient expressions, body language or choice of words a character expresses. Rule-based explanations for a character’s action will be given. Emotions or facial expressions will be explained in detail, and other examples will be given to allow the participant to generalize from the film scene to other social situations. An experimenter is present with the participant, helps understand the questions and feedback and can motivate and encourage the child throughout the training.

### Statistical methods

The planned sample size was calculated using a two-sided *t*-test (significance level *α* = 0.05) with a power 1−*β* = 80% based on a medium expected effect size of *d* = 0.6. Previous studies in ASD have shown similar or larger effect sizes for tDCS [71]. This effect requires a sample size of 45 patients per group (G*Power 3.1); assuming 10% drop-out, the total sample size will thus consist of 100 individuals randomized to tDCS or sham.

The primary analysis will be based on the intention-to-treat (ITT) set, including all randomized patients irrespective of the amount of treatment actually received or adherence to the intervention. A per-protocol (PP) analysis will be performed as sensitivity analysis with only those participants who received at least 9 out of 10 stimulation sessions without further relevant protocol deviations. In order to follow the intention-to-treat principle as closely as possible, all participants will be asked to participate in the end-of-treatment and follow-up assessment, even if they drop out of treatment, to minimize the amount of missing data.

We will test for a difference in the primary outcome measure, the change in SRS-16 item short form between post-treatment (T3) and baseline (T2), between the two study arms using a mixed-effects model repeated measures analysis with covariates centre, gender, age, IQ and the baseline value of the primary outcome (SRS-16 at T2). No unadjusted analyses are planned. Emphasis will be on the 95% confidence intervals of the effect size estimates; additionally, *p* values will be calculated. Secondary outcomes and the additional outcomes at the follow-up assessment (T4) will be analysed in a similar way. No subgroup analyses are planned, and any post hoc subgroup analyses would be considered exploratory. In this clinical trial, any missing values of the primary outcome variable have to be considered to be missing not at random (MNAR), in particular patients with very poor response to the intervention might be more likely to drop out of treatment and might be more likely not to provide data on the primary outcome (loss to follow-up). Thus, imputation strategies or analysis methods that rely on the missing at random (MAR) assumption could be anti-conservative. In the primary analysis of the primary endpoint, we will use all available measures of the primary outcome (SRS-16) at all time-points (in particular T2-5 and follow-up T4) in a mixed-effects model repeated measures analysis without imputation of missing values. Sensitivity analyses will be performed to investigate the potential impact of missing data, in particular, by performing a complete case analysis and a pre-specified conservative single imputation approach and by using multiple imputations. All analyses will be pre-specified in a statistical analysis plan. Statistical analyses will be performed using the statistical software IBM SPSS Statistics. SPSS will be used for descriptive statistics and all main analyses. There will be no interim analysis.

### Data collection and quality assurance

Data collection will be handled by experienced study personnel who received training in all study-related procedures, as well as good clinical practice related to the investigation of medical devices. A validated eCRF software will be used for data entry. Only authorized and trained personnel will get a password/username and are allowed to enter data. All entries and changes will be logged via an audit trail. Data management of clinical study data is handled by “Zentrum für Klinische Studien (ZKS) Kiel”. This includes eCRF construction, training and support of study sites regarding the eCRF system, query management, data cleaning and database lock. A validated study database system, Marvin (provided by XClinical), will be used. The clinical data entered into the eCRF will be stored on the XClinical servers in Nuremberg and Munich (Germany) and will be released to Frankfurt and other partners within the STIPED consortium for planned data analysis after the end of the study. Participants are informed about this procedure in the data protection paragraph of the informed consent forms. The data management plan (DMP) is issued by ZKS and describes all functions, processes and specifications for data collection, cleaning and validation. The trial monitoring will also be carried out by the ZKS, according to a pre-specified monitoring plan. Furthermore auditing procedures are specified in a separate audit plan, which encompasses auditing procedures for the whole trial as well as individual study sites regarding general quality assurance and monitoring activities, or, e.g. in case of repeated protocol-deviations.

### Documentation and evaluation of adverse events

Previous studies in children have rarely reported serious adverse events (SAEs) and tDCS-related side effects generally seem to be mostly mild and transient (such as, e.g. tingling, itching, redness and scalp discomfort [27]). In the current trial, investigators record all adverse events (AEs) in an Adverse Event Log provided in the eCRF including time of onset and intensity, causal relationship, action taken and outcome. SAEs are documented in a Serious Adverse Event Log and additionally must be reported to the sponsor immediately. The sponsor will perform all SAE-reporting to responsible authorities according to applicable national laws. A SOP on handling SAEs is provided for all trial sites by the sponsor. In Germany, quarterly safety reports listing all SAEs will be provided by the sponsor. In France and in Portugal, annual safety reports will be submitted.

A DSMB with three independent members is installed, who will monitor safety data in fixed time intervals. The first meeting will be held after the tenth patient has been included in the trial. When 50% of the participants have been recruited, the second meeting will take place. Additional meetings will be organized on demand, e.g. in case of emergency, an unscheduled meeting can be performed. If necessary, the DSMB has access to the unblinded data to judge whether the risk-benefit ratio changes throughout the study. If the board decides that the safety of participants is significantly endangered, they can recommend stopping the study.

### Continued treatment and medical care of subject after end of study

Patients who are under the care of the investigating sites will be able to continue their previous/ongoing treatment after the end of the study. The access to regular medical and/or psychological care is not altered by participation in the clinical study. Participants who were referred to the study from other sources are offered counselling for treatment options and will be supported in finding adequate treatment options.

### Further information on the trial

This trial is a part of the EU-Project STIPED (“Stimulation in Pediatrics” within Horizon2020). The Goethe University Frankfurt is the sponsor of the trial and is represented by Prof. Dr. Christine M. Freitag, who is also the coordinating investigator of StimAT. Further information on the consortium and other relevant aspects of the study conduct can be found in the supplement. The trial was first submitted to the German Federal Institute for Drugs and Medical Devices (Bundestinstitut für Arzneimittel und Medizinprodukte, BfArM, Germany) and a waiver of authorisation was granted; furthermore, the trial was evaluated and approved by the ethical committee of the Faculty of Medicine at Goethe University Frankfurt (Ethik-Kommission des Fachbereichs Medizin der Goethe-Universität Frankfurt am Main, Germany). The trial is referenced under EUDAMED No. CIV-18-01-022765.

## Discussion

The present trial is a double-blind, sham controlled parallel group phase-IIa pilot study of tDCS of the bilateral TPJ in children and adolescents with ASD. It will be the first study powered to detect a medium effect size by tDCS targeting a part of the “social brain”, thereby exploring a new treatment option for core social communication and interaction deficits in ASD. To test effect, feasibility and safety of the intervention, several points have been taken into consideration during development of the study protocol.

To date, only few studies have investigated tDCS as intervention method for ASD and there is a high variability between study designs. Most trials have focused on stimulating the DLPFC [21–23, 25, 26]. Only one study has previously examined the effect of tDCS over the TPJ. Despite a small sample size, Esse Wilson et al. [40] found tentative evidence that stimulation of the TPJ may improve verbal fluency and social skills in ASD. These results are similar to studies in healthy controls showing that tDCS stimulation of the TPJ can successfully improve social abilities such as perspective taking or emotion attribution [37–39]. All studies, including Esse Wilson et al. [40], have used large sponge or surface electrodes placed according to the international 10–20 system. While this approach is commonly used in tDCS research, it only allows for a relatively coarse localization of the target area and in turn relies on the widely distributed current flow effected by these electrodes. In the proposed study, we took a different approach by developing an optimized multi-channel montage with DTI-based definition of the target area in order to increase precision of the stimulation. While this is a novel approach for ASD samples, a systematic comparison of conventional and multi-channel montages for stimulating the right inferior frontal gyrus in children and adolescents with ADHD has previously shown that multi-channel tDCS has equivalent, if not better, response rates to stimulation and can successfully elicit changes in neurophysiological parameters [72].

Studies have also varied greatly in the number and duration of stimulation sessions, the applied stimulation intensity and use of concurrent tasks. Applied stimulation intensities ranged from 1 to 2 mA and duration from a single 30-min session up to 10 or more 20-min sessions over several days. Often no cognitive task was applied during stimulation, although the only study stimulating the TPJ applied a battery of social cognitive tasks concurrently to stimulation [40], as in the present study. Evidence from ADHD research indicates that repeated sessions lead to longer-lasting effects and that on-line stimulation during a cognitive task improves the effectiveness of stimulation [18]. Taken together, the parameters chosen for the current study are well within conventional standards of comparable studies.

To examine the effects of tDCS stimulation in ASD a variety of outcome measures targeting core symptoms, cognitive and neurophysiological changes have been chosen, yet to date no study has taken into account all of them simultaneously. In the current study, change in parent-rated social responsiveness from baseline to post-intervention was chosen as the primary clinical outcome measure. The SRS-16 was selected, because the instrument is a reliable, valid and economic way to describe parent-rated ASD symptom severity, which is also less susceptible to the influence of age, gender and verbal and non-verbal level of functioning of the ASD individual, and shows superior internal consistency compared to the long version [48]. It therefore allows to evaluate generalized improvements in every-day social communication and interaction abilities in ASD. Most tDCS studies have applied the autism treatment evaluation checklist (ATEC [73]); however, the SRS is better validated and is more frequently used in clinical trials of behavioural interventions for ASD (see, e.g. [9, 74]) and brain stimulation using repetitive transcranial magnetic stimulation [75–79].

Furthermore, evaluating the safety and tolerability of the tDCS intervention is another primary aim of the study. Safety evaluations are not only an essential part of investigating new treatment methods such as tDCS by phase-IIa trials, but also have a specific relevance for the ASD population. Individuals with ASD often experience sensory abnormalities [80, 81] which are described as “hypo- or hyper-reactivity to sensory input and unusual interests in sensory aspects of the environment” in domain B of the diagnostic criteria for ASD according to DSM-5 [1]. Consequently, individuals with ASD may experience different adverse events compared to the non-ASD population. To date, studies in participants with ASD have not reported an increased occurrence of adverse reactions to tDCS stimulation, but due to the limited amount of studies, especially in adolescents with ASD, a further systematic evaluation is needed.

Several secondary outcome measures were also chosen to describe the effect size of change in additional ASD symptoms, additional psychopathology and health-related quality of life. To date, no tDCS study in ASD has applied such an extensive battery of clinical outcome measures. Most studies only focus on few measures assessing core ASD symptomatology but neglect other psychopathology or health-related quality of life. Especially the latter has been discussed as an essential outcome, as improving quality of life should be an important objective of any intervention study in ASD [82]. By this broad approach of applying multiple clinical outcome measures, not only desired improvements will be examined, but possible undesired deterioration can also be monitored. This approach is also in line with the “net zero-sum model” [83] which posits that gain in one neural domain may lead to loss in another, and that therefore a broad spectrum of functions should be monitored. Also, the multiple outcome measures will allow us to describe specific change of the targeted social cognitive domain or more general change in psychopathology, cognition or neural function.

The aim to cover a broad spectrum of functions was taken into account in the choice of neuropsychological tasks as additional secondary outcome measures. These do not only encompass functions closely related to the TPJ, such as imitation or emotion recognition abilities, but also attention and executive function. This also represents an extension of previous tDCS research in ASD, where only tasks directly related to the functionality of the stimulated area were examined (see, e.g. [23, 40]).

EEG and functional MRI measures will be used to describe functional changes on the neural level, with a focus on investigating changes in TPJ activation and connectivity patterns. Previous studies mostly focused on EEG or fMRI resting state spectral and connectivity analyses [22, 25, 26] and did not look at neural correlates of task performance.

In addition to describing changes in behaviour, cognition and brain function induced by the intervention, the study also aims at exploring moderators of intervention effects. Almost all clinical trials in ASD have described a large variation of intervention effects within the treated sample, such as behavioural interventions [9]. Thus, identifying and characterizing children and adolescents who profit from tDCS may pave the way to an ultimately individualized recommendation for study participants to further pursue tDCS treatment. This is addressed in the current study by using neuroanatomical biomarkers to predict treatment response as an important step towards personalized treatment of ASD as a heterogeneous neurodevelopmental disorder. To our knowledge, no previous study has implemented neuroanatomical, or any other, biomarkers in order to analyse variability in response to tDCS in ASD.

An extensive evaluation of possible benefits and risks of the study was performed prior to the start of the study. A clear benefit of the study is the access to controlled and monitored tDCS treatment, which is not available as a standard treatment for ASD. Potential benefits for participants receiving real tDCS stimulation are improved social communication symptoms in ASD. The present study investigates tDCS as an add-on to a cognitive training focussing on highly relevant cognitive aspects underlying successful social communication. The benefits for all patients, regardless whether they are allocated to the tDCS or the sham condition, are participation in the cognitive training, which is provided independent of the stimulation (tDCS/sham). Cognitive trainings, e.g. of emotion recognition, have been studied in ASD before, but results have mostly shown that improvements can only be seen in proximal tasks evaluating the trained skill, while there is still little evidence of generalization to everyday life social interactions [84]. Combining a cognitive training with tDCS may serve to amplify the effects and therefore lead to improvements in more distal measures of social skills.

Regarding potential adverse effects, studies examining the safety aspects of tDCS in adult populations have found no adverse effects related to motor performance, the spectral characteristics of EEG or other clinical measures of brain function [85–87]. The levels of neuron-specific enolase, a sensitive marker of neuronal damage, were not increased after tDCS [88], and no pathological changes were observed in contrast-enhanced MRI or EEG [88, 89]. A comprehensive review on safety aspects of the tDCS was published by an International Safety Consortium [90], summarizing experimental (animal and human), clinical and computational studies which have been performed until 2016 in healthy subjects, patients and in theoretically vulnerable populations including children and the elderly; subjects with mood disorders, epilepsy, stroke and implants; and home users. Evidence from relevant animal and computational models indicates that brain injury by tDCS occurs at predicted brain current densities (6.3–13 A/m^2^) that are over an order of magnitude above those produced by conventional tDCS, such as used in the present clinical study (max. 2 mA). Regarding long-term safety aspects in children and adolescents, the longest follow-up period was 1.5 years and no adverse responses were reported [27, 91]. Also regarding epilepsy, the majority of studies in both adults and children showed that tDCS does not elicit epileptic seizures or provoke epileptic EEG activity either in healthy subjects [51] or in children with known epilepsy [90, 92]. Only in one single case an epileptic seizure was observed during a tDCS session conducted to treat epilepsy in a child. Systematic investigations of AEs to tDCS in different age and subject groups show that participants tolerated the stimulation well with a low rate of AEs during and after both real stimulation and application of sham [93–96]. No serious adverse effects were seen in young patients, even after tDCS applied with a higher than usual current density (0.497 mA/cm^2^) and/or repeated over several days [20, 95, 97, 98]. Thus, we conclude that potential benefits outweigh the risks posed by participating in the clinical trial.

If the current evaluation of tDCS over the bilateral TPJ shows positive changes in ASD-related behaviour and confirms good safety and tolerability of the method, tDCS may represent a new, cost-efficient and easily applied therapy option for the disorder. Detailed analysis of the collected data will also enable us to further optimize parameters of the stimulation and to develop personalized treatment approaches based on biomarkers. This may however only be a first step. The easy application of tDCS also opens up further possibilities, as similar stimulation paradigms may be applied in other settings. For example, the application at home could be a promising cost-efficient way to offer additional intervention for autistic children, which is even more convenient for affected individuals and their families. Furthermore, the concurrent application of tDCS could be an approach to amplify the positive effects of behavioural therapy, which could be implemented in a clinical setting.

### Limitations

Even though the design of the proposed study was carefully considered, there are several limitations to the study. Firstly, the proposed design only entails a relatively short follow-up period after the intervention of 4 weeks. In previous studies, this period has varied greatly, and the last time point of observation has ranged from directly after stimulation [40] to up to 7 months’ follow-up [22]. Recent studies however also suggest that effects of combined cognitive training and tDCS stimulation can be stable for up to 1 year [99].

Furthermore, we chose a multi-channel montage to target the bilateral TPJ. We have outlined the advantages of this approach above. However, it could be argued that individualized montages, e.g. based on individual fMRI or DTI data, may be even better to increase accuracy in the stimulation of the target brain area. However, at the time of study conceptualization, generating those individual montages still had a relatively high cost with respect to computational effort and time needed and was therefore not implemented. Systematic comparisons of different stimulation montages are an important aspect for future research.

Another potential problem of the study design is the unknown re-test reliability and possible training effects of some outcome measures such as the neurocognitive and EEG tasks. A recent systematic evaluation of the re-test reliability of EEG markers in an autistic sample has shown that inter-class correlation for repeated assessment of EEG measures can range from, e.g. 0.83 for resting-state slope to − 0.09 for the N2 in a biological motion paradigm [100]. Unfortunately, the reliability of the tasks which are implemented in the current study is yet unknown. This was also a reason why these measures were not chosen as primary outcome. In addition, where possible, measures were taken to mitigate the potential problems of repeated assessments, e.g. by implementing three parallel versions of the intention tasks for the three measurement time points.

Also, only high functioning individuals with ASD are eligible for study participation. The relatively high cognitive demand of the training, as well as the extensive battery of outcome measures, makes it impossible for intellectually disabled individuals with ASD to participate. This means that the intervention is only suited for part of the ASD population. However, if the combination of tDCS and cognitive training prove effective in the current study population, it may be worthwhile to adapt the procedures in order to also make the intervention available to intellectually disabled individuals with ASD.

## Trial status

The current description of the trial refers to study protocol version 1.2 from 24 May 2019, i.e. the second amended version, of the study protocol. Recruitment for the trial started in March 2019 and is ongoing. It will be completed approximately in October 2021.

## Supplementary Information

**Additional file 1.**

## Data Availability

Data is only available for members of the STIPED consortium in accordance with the STIPED memorandum of understanding. Other materials such as information regarding EEG tasks and the social cognitive training battery or stimulation montage are available upon request. Model-informed consent forms (in German, French and Portuguese) are available from the corresponding author on request. Results of the trial will be published in appropriate peer-reviewed journals.

## Abbreviations

ABC: Aberrant Behavior Checklist
ADHD: Attention deficit hyperactivity disorder
ADI-R: Autism Diagnostic Interview-revised
ADOS-2: Autism Diagnostic Observation Schedule-2
AE: Adverse event
ASD: Autism spectrum disorder
ATEC: Autism treatment evaluation checklist
CANTAB: Cambridge Neuropsychological Test Automated Battery
CBCL: Child Behaviour Checklist
CCC-2: Children’s Communication Checklist-2
CRF: Case report form
DC: Direct current
DLPFC: Dorsolateral prefrontal cortex
DRKS: German Clinical Trials Register
DSM-5: Diagnostic and Statistical Manual of Mental Disorders, 5th edition
DTI: Diffusion tensor imaging
eCRF: Electronic case report form
EEG: Electroencephalogram
ERP: Event-related potential
fMRI: Functional magnetic resonance imaging
GCP: Good Clinical Practice
IMIS: Institute of Medical Informatics and Statistics Kiel
IQ: Intelligence quotient
KIDSCREEN 27: Health-Related Quality of Life Questionnaire for Children and Adolescents
K-SADS-PL: Revised Schedule for Affective Disorders and Schizophrenia for School-Age Children: Present and Lifetime Version
mA: Milliampere
MAR: Missing at random
MNAR: Missing not at random
MRI: Magnetic resonance imaging
PDS: Pubertal Development Scale
RBS-R: Repetitive Behaviour Scale-revised
SADE: Serious adverse device effect
SAE: Serious adverse event
SRS: Social Responsiveness Scale
SRS-16: Social Responsiveness Scale: 16 item short form
SSRI: Selective serotonin reuptake inhibitors
STIPED: Stimulation in Pediatrics consortium
TPJ: Tempo-parietal junction
tCS: Transcranial stimulation
tDCS: Transcranial direct current stimulation
TES: Transcranial electric stimulation
ZKS: Zentrum für Klinische Studien (Center for Clinical Trials) Kiel
