# Supplementary material for: Phase-IIa randomized, double-blind, sham-controlled, parallel group trial on anodal transcranial direct current stimulation (tDCS) over the left and right tempo-parietal junction in autism spectrum disorder—StimAT: study protocol for a clinical trial
Source: Trials. 2021 Apr 6;22:248. doi: 10.1186/s13063-021-05172-1 (PMC8025356; doi:10.1186/s13063-021-05172-1)
Supplement: Supplementary file 1 — Additional file 1. [file 13063_2021_5172_MOESM1_ESM.pdf]

Supplement to “Phase-IIa randomized, double-blind, sham-controlled, parallel group trial on anodal tDCS over the left and right tempo-parietal junction in Autism Spectrum Disorder – StimAT”

List of other diagnostic procedures and measures

- Semi-structured medical history interview, screening for brain related disorders, tDCS safety criteria. A clinical interview with the parents, assessing information on pregnancy, birth parameters, early development, educational achievement, previous interventions, medical, neurological and psychiatric disorders, previous and current medication, concurrent neuro-feedback or stimulation therapy and parental education status.
- Screening for vision and hearing impairments using the Broken Ring vision chart, also known as Landolt Rings, to test for visual acuity and the Weber and Rinne tuning fork test.
- MRI safety criteria questionnaire, assessing whether participants have any contraindications for undergoing MRI measurements e.g. have had a serious brain injury, or carry metal implants.
- Structured interview K-SADS-PL (Kaufman et al. 1997) which is a semi-structured interview with the parents on DSM-IV TR based criteria for most psychiatric disorders. A version adapted to DSM-5 covering all psychiatric disorders which lead to exclusion from the study as well as all relevant comorbid disorders will be used.
- The autism diagnostic observation schedule (ADOS-2) is a structured behavioural observation measure eliciting core autism symptoms in the domains of social interaction and stereotyped and repetitive behaviour (Lord et al. 2015). Within the study, existing ADOS-2 results can be used if obtained within 3 years prior to inclusion at the same centre.
- Structured interview: ADI-R: The Autism Diagnostic Interview – revised (Rutter et al. 2003) is a semi-structured interview with the parents assessing information on early development and autism specific behaviour. Together with the ADOS-2 (see above), information from the ADI-R is used to establish the diagnosis of Autism Spectrum Disorder according to DSM-5. Within the study, existing ADI-R results can be used, if they were obtained at any point previously at the same centre. If a new diagnosis has to be established, the ADI-R algorithm items will be obtained within this study.
- Intelligence test: To measure IQ, we will use the Vocabulary and Picture Completion subscales from the third editions of the Wechsler Intelligence Scale for Children (Wechsler 1991) / Adults (Wechsler 1997). The Wechsler Intelligence Scales are one of the most commonly used instruments for intelligence testing; however, completing all subtests would require too much time. By utilizing only the Vocabulary and Picture Completion subscales, we ensure that two relevant factors of intelligence, verbal comprehension and perceptual organization (assessing Verbal IQ and Performance IQ, respectively), are covered in the test, and while keeping the time

needed to conduct the test at a minimum. Within the study, existing IQ results can be used if obtained within 1 year prior to inclusion at the same center.

- Edinburgh Handedness Inventory (EHI):The EHI is a short scale used to assess the dominance of a person's right or left hand in everyday activities and is widely used in research (Oldfield 1971).
- Pubertal Development Scale (PDS) allows measuring the pubertal stage without visual inspection by a clinician (Carskadon und Acebo 1993). The single sum score differentiates five different pubertal stages for boys and girls (cronbach's alpha:.59 to .71).
- Urine pregnancy test in female participants: Female participants will provide a urine sample for a pregnancy testing. Standard laboratory procedure at each site will be implemented.

Questionnaire on expectations and concerns of parents and participants towards tDCS: In order to investigate motivation/intention towards application of tDCS in paediatric population, a special questionnaire was developed based on the well-established theory of planned behaviour (TPB) (Ajzen 2011). Research supports the usefulness of the TPB in the prediction of behavioral intention and performance for a wide variety of health behaviours and behavioural interventions (McEachan et al. 2011). The TPB enables not only an assessment of the strength of motivation/behavioural intention (for example towards application of tDCS) but also a differentiation of the mentioned constructs, which is essential for explaining attitudinal changes and needs to be considered to improve adherence to a specific intervention (McDermott et al. 2016).

Reimbursement of participants

Participants will receive no remuneration for visits involving the socio-cognitive training with intervention (sham or real tDCS stimulation, T2_1-T2-10). In Germany, participants are reimbursed for screening (T1), baseline (T2), post-intervention (T3) and follow-up (T4) with 20 € per visit. For those participants who are safe to complete MRI visits, a further 10€ per MRI visit will be paid. This allows for a maximum total of 100€ per participant, or 80€ if MRI visits are not attended. In France, to acknowledge the time and effort spent by the participants and their families, they will receive 100€. In Coimbra, it is not allowed to financially reimburse study participants, thus, they will not receive any payment for their participation in the study. However, to acknowledge the time and effort spent by the families, participants and their families are reimbursed for costs with traveling, meals and accommodation through payment of daily allowances.

Protocol versions and amendments/ ethics

The study will be conducted in accordance with the study protocol, the principles of the Declaration of Helsinki, as well as Good Clinical Practice, relevant national laws and applicable federal and local regulatory requirements for data privacy protection. All essential documents will be archived at each participating site. The protocol and local Informed Consent (IC) forms must be reviewed and approved in writing by an Independent Ethical Committee (IEC) and the federal authority prior to the initiation and subject recruitment. Each of the IEC and the federal authorities must be notified of all subsequent protocol amendments. Any change to this protocol will be documented in a protocol amendment and agreed upon by the sponsor and the trial statistician before its implementation. Protocol amendments will be submitted for notification to the EC and competent authority, in accordance with local regulations. An approval by the EC is required for a substantial amendment, e.g. one which could affect the safety of the subjects, or which entails a change to the scope/design of the trial.

The protocol must be strictly adhered to. Each deviation has to be documented and justified in written form. The current description of the trial refers to study protocol version 1.2 from 24.05.2019, i.e. the second amended version, of the study protocol. Any changes to the protocol will be documented in a protocol amendment and agreed upon by the sponsor and the trial statistician before its implementation. Protocol amendments will be submitted to the competent authorities and ethics committees for approval, in accordance with local regulations. Once amendments are approved, they will be forwarded to study sites and respective investigators in order to be filed in the investigator site file.

Responsibilities & consortium

This trial is a part of the EU-Project STIPED (“Stimulation in Paediatrics” within Horizon2020). Prof. Dr. Christine M. Freitag is the coordinating investigator of StimAT and also the sponsor’s representative for the Goethe University Frankfurt (Theodor-W.-Adorno Platz 6, 60323 Frankfurt am Main, Germany) for this investigator-initiated trial. Prof. Dr. Astrid Dempfle (Institute of Medical Informatics and Statistics, Christian-Albrechts-University Kiel) is the statisticaian. Data Management is done by the „Zentrum für Klinische Studien (ZKS) Kiel“.

The study is conducted at 4 clinical sites: (1) the Department of Child and Adolescent Psychiatry, Psychosomatics and Psychotherapy and Autism Research and Intervention Center of Excellence at the University Hospital Frankfurt, Goethe University (Investigator: Prof. Dr. C. M. Freitag), (2) the Department of Child and Adolescent Psychiatry, Psychosomatics and Psychotherapy at Bethel Protestant Hospital in Bielefeld, Germany (Investigator: Prof. Dr. M. Siniatchkin), (3) the ICNAS, Centro Clínico Académico, Universidade de Coimbra, Portugal (Investigator: Prof. Dr. M. Castelo-Branco), and

(4) the Centre Hospitalier Regional Universitaire de Tours, Tours, France (Investigator: Prof. Dr. F. Bonnet-Brihault). Prof. Dr. Michael Nitsche, Prof. Dr. Christian Plewnia, Prof. Dr. André Scherag are members of the DSMB.

The trial steering committee consists of the principal investigators, the statistician and the data management team. It meets biannually to discuss organisational matters of trial conduct.

Further information can be requested directly from the coordinating investigator regarding:

- Informed consent: further information and example consent forms are available upon request
- Socio-cognitive training battery
- Trial conduct (insurance, safety, reporting, data-protection etc.)

References

Ajzen, Icek (2011): The theory of planned behaviour: reactions and reflections: Taylor & Francis.

Carskadon, Mary A.; Acebo, Christine (1993): A self-administered rating scale for pubertal development. In: *Journal of Adolescent Health* 14 (3), S. 190–195.

Kaufman, J.; Birmaher, B.; Brent, D.; Rao, U.; Flynn, C.; Moreci, P. et al. (1997): Schedule for Affective Disorders and Schizophrenia for School-Age Children-Present and Lifetime Version (K-SADS-PL): initial reliability and validity data. In: *Journal of the American Academy of Child and Adolescent Psychiatry* 36 (7), S. 980–988. DOI: 10.1097/00004583-199707000-00021.

Lord, C.; Rutter, M.; DiLavore, P. C.; Risi, S.; Gotham, K.; Bishop, S. L.; Schedule, ADOS Autism Diagnostic Observation (2015): ADOS-2. In: *Manual (Part I): Modules*, S. 1–4.

McDermott, Máirtín S.; Oliver, Madalyn; Iverson, Don; Sharma, Rajeev (2016): Effective techniques for changing physical activity and healthy eating intentions and behaviour: A systematic review and meta-analysis. In: *British journal of health psychology* 21 (4), S. 827–841. DOI: 10.1111/bjhp.12199.

McEachan, Rosemary Robin Charlotte; Conner, Mark; Taylor, Natalie Jayne; Lawton, Rebecca Jane (2011): Prospective prediction of health-related behaviours with the Theory of Planned Behaviour: a meta-analysis. In: *Health Psychology Review* 5 (2), S. 97–144. DOI: 10.1080/17437199.2010.521684.

Oldfield, R. C. (1971): The assessment and analysis of handedness: The Edinburgh inventory. In: *Neuropsychologia* 9 (1), S. 97–113. DOI: 10.1016/0028-3932(71)90067-4.

Rutter, Michael; Le Couteur, A.; Lord, C. (2003): Autism diagnostic interview-revised. In: *Los Angeles, CA: Western Psychological Services* 29, S. 30.

Wechsler, David (1997): WAiS-iii: Psychological Corporation San Antonio, TX.
